# Supplementary material for: A Critical View of the Application of the APEX Software (Aqueous Photochemistry of Environmentally-Occurring Xenobiotics) to Predict Photoreaction Kinetics in Surface Freshwaters
Source: Molecules. 2019 Dec 18;25(1):9. doi: 10.3390/molecules25010009 (PMC7017383; doi:10.3390/molecules25010009)
Supplement: Supplementary file 1 [file molecules-25-00009-s001.zip › 3.2.4_gcc-4.4.0.zip!/3.2.4_gcc-4.4.0/3.2.4_gcc-4.4.0/doc/octave/html/interpreter/Linear-Algebra.html]

Linear Algebra - Untitled


Next: Nonlinear Equations,
Previous: Arithmetic,
Up: Top


---

## 18 Linear Algebra

This chapter documents the linear algebra functions of Octave.
Reference material for many of these functions may be found in
Golub and Van Loan, Matrix Computations, 2nd Ed., Johns Hopkins,
1989, and in the lapack Users' Guide, SIAM, 1992.

- Techniques used for Linear Algebra- Basic Matrix Functions- Matrix Factorizations- Functions of a Matrix- Specialized Solvers
